# Upstream-binding factor is sequestered into herpes simplex virus type 1 replication compartments

**DOI:** 10.1099/vir.0.006353-0

**Published:** 2009-01

**Authors:** Nigel D. Stow, Vanessa C. Evans, David A. Matthews

**Affiliations:** 1 MRC Virology Unit, Institute of Virology, Church Street, Glasgow G11 5JR, UK; 2 Department of Cellular and Molecular Medicine, School of Medical Sciences, University of Bristol, Bristol BS8 1TD, UK

## Abstract

Previous reports have shown that adenovirus recruits nucleolar protein upstream-binding factor (UBF) into adenovirus DNA replication centres. Here, we report that despite having a different mode of viral DNA replication, herpes simplex virus type 1 (HSV-1) also recruits UBF into viral DNA replication centres. Moreover, as with adenovirus, enhanced green fluorescent protein-tagged fusion proteins of UBF inhibit viral DNA replication. We propose that UBF is recruited to the replication compartments to aid replication of HSV-1 DNA. In addition, this is a further example of the role of nucleolar components in viral life cycles.

The nucleolus is a dynamic multi-protein structure whose primary role is the synthesis of rRNA followed by processing and incorporation of rRNA into functional ribosomes for export to the cytoplasm. However, in the past few decades it has become clear that the nucleolus plays a role in very diverse aspects of the cellular life cycle from cell cycle control to innate immune responses (Boisvert *et al.*, 2007; Pederson, 1998). A natural consequence of this is that a large number of viruses have also been shown to interact with the nucleolus on many different levels (Hiscox, 2007). We recently showed that adenovirus, which has a linear double-stranded DNA genome, interacts with the nucleolar component called upstream-binding factor (UBF). UBF is normally associated with rDNA at the rRNA promoters and plays a role in the recruitment of RNA pol I to rDNA, which ultimately influences the rate of rRNA synthesis (Grummt, 2003; McStay & Grummt, 2008). During adenovirus replication this cellular protein is sequestered from the nucleolus into adenoviral DNA replication centres (Lawrence *et al.*, 2006). Although UBF has a strong preference for rDNA it does not show sequence specificity and it is believed that DNA structure rather than sequence is more important in the sequestration of UBF (Copenhaver *et al.*, 1994; Kuhn *et al.*, 1994). Indeed, we proposed that UBF in adenovirus-infected cells was attracted to the replicating ends of the viral DNA, perhaps due to the formation of pan-handle structures that are thought to play a role in adenovirus DNA replication. We speculated that other DNA viruses might also interact with this cellular protein and therefore examined herpes simplex virus type 1 (HSV-1). In contrast to adenovirus, which uses a linear genome as a template for viral DNA replication, HSV-1 initially circularizes its viral DNA to provide a template for viral DNA replication (Boehmer & Lehman, 1997; de Jong *et al.*, 2003; Liu *et al.*, 2003; Strang & Stow, 2005). In addition, there is a strong body of literature showing that HSV-1 interacts with the host cell nucleolus on many levels (Ahmed & Fraser, 2001; Bertrand & Pearson, 2008; Besse & Puvion-Dutilleul, 1996; Calle *et al.*, 2008; Cheng *et al.*, 2002; Lymberopoulos & Pearson, 2007; Mears *et al.*, 1995; Puvion-Dutilleul *et al.*, 1986; Roller & Roizman, 1992). We hypothesized that UBF might be associated with the viral DNA replication compartments as defined by the presence of the HSV-1 single-stranded DNA-binding protein, ICP8. This essential protein, which serves as an excellent marker for viral DNA replication, has been the subject of a recent proteomic analysis revealing a number of interacting partners, including chromatin remodelling factors and DNA damage repair proteins (Taylor & Knipe, 2004).

HeLa cells grown on glass coverslips were infected with HSV-1 at an m.o.i. of 5 p.f.u. per cell and fixed for immunofluorescence using 4 % formaldehyde at 7 h post-infection (p.i.) by which time significant DNA replication is well under way, but little progeny virus will have been produced. The fixation and solublization process was similar to that used previously (Lawrence *et al.*, 2006) with the exception of the addition of normal human IgG (Sigma) to block non-specific binding of gE to the Fc receptors of the primary and secondary antibodies. We used both a commercially available anti-UBF antibody (H300; Santa Cruz) and one raised in sheep (Mais *et al.*, 2005) that was affinity purified (kindly provided by B. McStay, University of Dundee, UK). In addition, we used a mouse monoclonal antibody (mAb) to ICP8 (mAb 7381; Everett *et al.*, 2004) to determine which cells were infected and the relationship, if any, to UBF. As shown in Fig. 1(a) infected cells do demonstrate sequestration of some, but not all, UBF into ICP8-rich centres at 7 h p.i. In normal interphase cells it is well established that all the cellular UBF is located within the nucleolus (e.g. see Fig. 1a[Fig f1] from Lawrence *et al.*, 2006).

To compare, we also examined the location of endogenous B23.1 using a mAb (Zymed). This cellular protein (also known as nucleophosmin or NPM) is highly abundant in the host cell nucleolus, is involved in rRNA processing and is a standard reference antigen for the nucleolus. Indeed, Calle *et al.* (2008) have shown that B23.1 is more resistant to removal from the nucleolus than other nucleolar antigens such as nucleolin, although by 14 h p.i., in their work, B23.1 is clearly distributed throughout the nucleus. Fig. 1(b) shows that at 7 h p.i., UBF is predominantly localized both within the nucleolus (as delineated by B23.1) and in centres external to the nucleolus (shown by arrows). By 24 h p.i. (corresponding to the end of the lytic cycle), endogenous UBF is present mainly in compact dots, whereas B23.1 is no longer contained within discrete centres as has been reported previously (Calle *et al.*, 2008). This may indicate that UBF plays distinct roles at early and late times, since by 24 h the replication compartments will be quite large. The role of nucleolin in HSV-1 replication has recently been reported and partial co-localization of some of the available nucleolin with ICP8 was observed (Calle *et al.*, 2008). We did not examine nucleolin in this report, but we did obtain comparable results to Calle *et al.* (2008) for B23.1, which suggest that B23.1 is not markedly affected until after 8–14 h p.i. However, by 24 h p.i. there has been significant condensation of the nuclear chromatin with presumably extensive disruption of nuclear architecture and as such it is difficult to evaluate the effects of HSV-1 on B23.1. Notably, at 7 h p.i., we observed discrete circles of UBF within the nuclei of some cells (e.g. Fig. 1a[Fig f1]); whilst this phenomenon was common (approx. 25 % of cells), it was not universal and appeared to be restricted to this time (data not shown). We do not know what these structures represent at this time, but we have not observed them in either uninfected cells or adenovirus-infected cells.

Building on this we examined whether enhanced green fluorescent protein (EGFP)-tagged variants of UBF were also sequestered into the ICP8-rich centres. We therefore used selected deletion mutants of UBF that we had previously shown could be used to examine the role of UBF in adenovirus DNA replication (Lawrence *et al.*, 2006). Cells were transfected with various plasmids expressing UBF and deletion mutants fused to EGFP. After approximately 16 h, we then infected the cells with HSV-1 at an m.o.i. of about 5 p.f.u. per cell. Fig. 1(c) shows that full-length UBF–EGFP along with clones 1–572–EGFP and 1–372–EGFP were sequestered into ICP8-rich centres. This provides antibody independent confirmation that UBF is localized to ICP8-rich centres during infection. Indeed, with the EGFP fusion proteins it was sometimes difficult to detect the fusion protein anywhere other than within the replication centres. Frequently, the EGFP fusion protein would concentrate in discrete dots within the ICP8-rich centres. However, this pattern of co-localization was not prominent when using 1–272–EGFP, where UBF appeared to be mainly just outside ICP8-rich centres or with 1–102–EGFP which showed only a very faint association with ICP8-rich centres. This is consistent with our data for adenovirus-infected cells, which also showed that the first 372 aa of UBF are essential for the pattern of co-localization to match that of full-length UBF (Lawrence *et al.*, 2006). Full-length UBF contains 6 HMG box domains arranged in tandem, along with a transactivation domain and a dimerization domain (Fig. 1d[Fig f1]). Our data indicate that a minimum of the dimerization domain and 3 HMG boxes are needed for this protein to be sequestered into HSV-1 DNA replication centres. The circles of UBF seen when using antisera to endogenous UBF (Fig. 1a[Fig f1]) were not formed by EGFP–UBF fusion proteins, which may indicate that the fusion protein is unable to participate in these structures.

We also examined how RNA pol I and RNA synthesis were affected by this sequestration. In normal, uninfected cells, RNA pol I, UBF and the bulk of rRNA synthesis is normally localized together within the nucleolus (e.g. see Fig. 8 of Lawrence *et al.*, 2006). We determined the location of RNA pol I and RNA synthesis in HSV-1-infected cells using fluorouridine (FU) labelling for 15 min as described previously (Lawrence *et al.*, 2006). During adenoviral infection we observed that almost all the UBF was sequestered into virus replication centres leaving RNA pol I inside the nucleoli, where it continued to synthesize rRNA. In the case of HSV-1 it appeared that, as with adenovirus, UBF can be sequestered independently of RNA pol I (Fig. 2a[Fig f2]). In addition, we were able to readily detect RNA synthesis associated with RNA pol I. However, what is different in the case of HSV-1 infection is that not all of UBF is sequestered from the nucleoli. This may indicate that the phosphorylation status of UBF, for example, is important for UBF to participate in HSV-1 replication. Alternatively, it may simply mean that the HSV-1 replication compartments do not require all the UBF available within a cell.

Western blot analysis indicated that there was little reduction of levels of both isoforms of UBF (called UBF1 and UBF2) over time (Fig. 3a[Fig f3]). However, we did see a decline in the levels of UBF1 phosphorylated at ser 388 (this region is missing from UBF2). This phosphorylation event is needed to recruit RNA pol I and resume rRNA synthesis after completion of the cell cycle (Voit & Grummt, 2001). This may indicate that the dissociation of UBF from RNA pol I during HSV-1 infection is directly or indirectly linked to changes in the phosphorylation status of UBF. However, we did not observe significant changes in phosphorylation of UBF until 24 h p.i., whereas significant uncoupling of UBF from pol I does occur by 7 h p.i. This change in phosphorylation was not observed during adenovirus infection which supports the notion that UBF can be uncoupled from pol I without affecting its phosphorylation status, a hypothesis supported by recent reports that acetylation of UBF may also play a role (Meraner *et al.*, 2006). We also noted that phosphorylated UBF is degraded during infection; the functional significance of this has yet to be determined.

Finally, we wished to determine if there was a functional relationship between UBF and HSV-1 DNA replication. In our previous report (Lawrence *et al.*, 2006) we noted that expression of the EGFP fusion of full-length UBF, but not of UBF residues 1–192, had a notable dominant-negative inhibitory effect on adenovirus DNA replication. To determine if UBF fusion proteins could similarly affect HSV-1 DNA synthesis and growth we used two previously described plasmid-based transfection assays (Stow *et al.*, 1993). In the first assay, plasmids encoding the seven HSV-1 DNA replication proteins were co-transfected with a plasmid, pSl, containing a functional viral origin of DNA replication, in the presence or absence of plasmids encoding the UBF fusion proteins. Replicated pSl DNA was detected by Southern blot hybridization following cleavage of cellular DNA with *Eco*RI and *Dpn*I. Fig. 3(b) shows that co-transfection of either UBF–EGFP, 1–572–EGFP or l–362–EGFP noticeably inhibited viral DNA replication, whereas shorter truncations did not. This corresponded well with the ability of the three larger clones to associate with ICP8-rich centres in a manner similar to endogenous UBF. Most marked inhibition was observed with pE9CT, which encodes the DNA-binding domain of the HSV-1 origin-binding protein and has previously been shown to be a potent dominant-negative inhibitor of viral DNA synthesis (Stow *et al.*, 1993). In the second assay, we explored the ability of the UBF constructs to inhibit the ability of transfected HSV-1 DNA to generate plaques (Fig. 3c[Fig f3]). Briefly, infectious HSV-1 DNA was co-transfected with one of the plasmids expressing either full-length UBF–EGFP or a deletion mutant as described previously (Stow *et al.*, 1993). After 3 days, the number of plaques generated from each co-transfection was counted. In this assay, UBF–EGFP, 1–572–EGFP and pE9CT had the greatest inhibitory effects on the production of infectious progeny and almost abolished plaque formation. Smaller inhibitory effects were noted with the other UBF constructs although the mechanism for this is not clear. It was previously noted that the plaque assay was more sensitive than the origin-dependent DNA synthesis assay at detecting inhibitory effects of overexpressed proteins (Stow *et al.*, 1993).

Taken together, our data show that UBF is sequestered into viral DNA replication centres, that (as with adenovirus) UBF–EGFP fusion proteins inhibit viral DNA replication and that this sequestration does not directly affect rRNA production or the localization of RNA pol I. We believe this is strong evidence that UBF is a co-factor in the replication of HSV-1 DNA. We propose that, as with adenovirus, UBF is attracted to a structural feature of replicating HSV-1 DNA. Further work will uncover how UBF interacts with the replication machinery and what roles it may serve as the infection progresses. This paper adds UBF to the growing list of interactions between the nucleolus and various herpes viruses. In addition, it reinforces the concept that proteins in the nucleolus may play important roles during many viral life cycles.

## Figures and Tables

**Fig. 1. f1:**
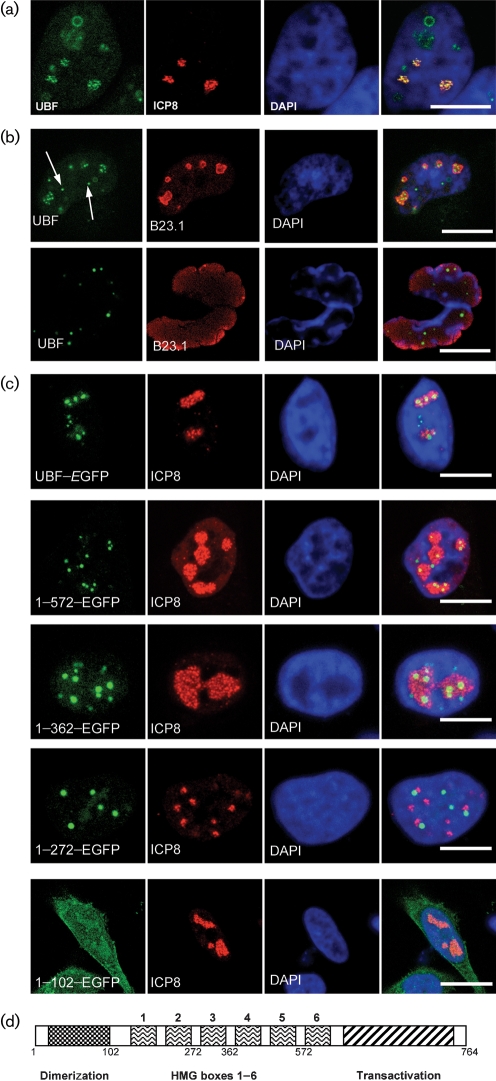
UBF is enriched in viral replication centres. (a) Cells infected with HSV-1 and probed with ICP8 antibodies at 7 h p.i. The location of UBF is shown in green, ICP8 protein is in red and the extent of the cell nucleus is revealed by staining with DAPI in blue. (b) Infected cells at 7 h p.i. (top row) and 24 h p.i. (bottom row). Arrows point to sites of extra nucleolar accumulation of UBF. The location of UBF is in green, B23.1 is in red and DAPI is in blue. All images are of a single focal plane approximately 0.3 μm in depth. Bar, 10 μm. (c) Location of a number of UBF–EGFP fusion proteins in HeLa cells at 7 h p.i. In each case, the plasmid indicated had been transfected into the cells approximately 15 h prior to infection. The EGFP fusion is shown in green, ICP8 is in red and DAPI is in blue. UBF–EGFP contains the full-length protein. (d) Schematic diagram of UBF. The drawing indicates the relative location of the 6 HMG boxes (numbered 1–6), the dimerization domain (marked with checked fill) and the transactivation domain (marked with diagonal lines). In addition, below the diagram the approximate locations of the C-terminal amino acids of the deletion mutants used in this study (full-length UBF is 764 aa) are indicated.

**Fig. 2. f2:**
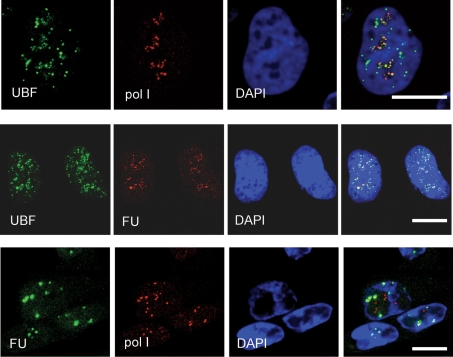
HSV-1 infection causes UBF to separate from RNA pol I and the major sites of RNA synthesis. HSV-1-infected cells at 7 h p.i., where the top row shows endogenous UBF (green), RNA pol I (red) and DAPI (blue). Similarly infected cells are shown in the middle row, endogenous UBF (green), RNA synthesis (detected by FU labelling for 20 min; red) and DAPI (blue) are shown. Similarly infected cells are also shown in the bottom row, where the location of FU (green), RNA pol I (red) and DAPI (blue) are shown. All images are of a single focal plane approximately 0.3 μm in depth. Bar, 10 μm.

**Fig. 3. f3:**
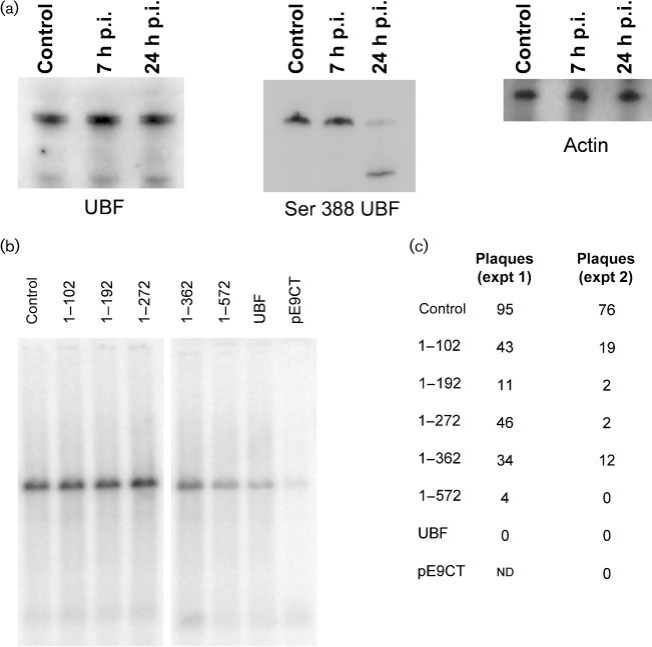
The effects of HSV-1 infection on UBF and the effects of UBF fusion proteins on HSV-1 replication. In (a) the levels of total UBF and phosporylated UBF were assessed by Western blotting. On the left is a Western analysis of cells harvested at 7 and 24 h p.i., showing levels of total UBF (both isoforms) by using an affinity purified anti-UBF serum raised in sheep. The same samples were analysed for phosphorylated ser 388 UBF. Also shown is a control Western blot using antisera to actin as a loading control. (b) Shows the effect of expression of UBF–EGFP fusion proteins on HSV-1 origin-dependent DNA synthesis in the presence of the indicated UBF fusion proteins or pE9CT. The position of the replicated pS1 molecules are indicated by the arrow. (c) Shows the effects of the indicated plasmids on the ability of infectious HSV-1 DNA to generate plaques in a co-transfection assay.
